# Effects of a Bentonite Clay Product and a Preservative Blend on Ileal and Fecal Nutrient Digestibility in Pigs Fed Wheat Naturally Contaminated with Deoxynivalenol

**DOI:** 10.3390/ani13243752

**Published:** 2023-12-05

**Authors:** Seung Youp Shin, Seung Bin Yoo, Yoon Soo Song, Noa Park, Beob Gyun Kim

**Affiliations:** Department of Animal Science, Konkuk University, Seoul 05029, Republic of Korea; shinsy02@konkuk.ac.kr (S.Y.S.); seungbinyoo0316@gmail.com (S.B.Y.); sang8sys@konkuk.ac.kr (Y.S.S.); parknoa0227@gmail.com (N.P.)

**Keywords:** deoxynivalenol, digestibility, feed additive, swine

## Abstract

**Simple Summary:**

Deoxynivalenol is a secondary metabolite produced by fungal activity which has detrimental effects on nutrient digestibility and growth performance in pigs. These metabolites are found in the grain feeds such as barley, corn, and wheat, which are major ingredients in pig diets. To mitigate the negative effects of deoxynivalenol, clay products are often supplemented in pig diets. However, clay products potentially bind nutrients, including minerals and amino acids, consequently decreasing the digestibility of these essential nutrients. On the other hand, a preservative blend product containing antioxidants, microorganisms, vitamins, and amino acids was reported to restore the impaired growth performance by deoxynivalenol. In the present study, sodium digestibility was decreased by dietary deoxynivalenol but restored with the supplementation of the preservative blend product. Zinc digestibility was increased by dietary deoxynivalenol but decreased when bentonite was added to the deoxynivalenol-contaminated diet. The digestibility of some amino acids was decreased by supplemental bentonite. Overall, the addition of the preservation blend product improved some mineral digestibility whereas the addition of bentonite decreased the digestibility of some minerals and amino acids.

**Abstract:**

The objectives were to determine the effects of dietary deoxynivalenol (DON) on apparent ileal digestibility (AID) of nutrients and to evaluate the efficacy of a bentonite (BEN) and a preservative blend (PB) product for alleviating DON effects on the nutrient digestibility of pigs. Twelve crossbred barrows with an initial body weight of 69.4 kg (standard deviation = 3.5) equipped with a T-cannula in the distal ileum were allotted a triplicated 4 × 2 incomplete Latin square design with four dietary treatments and two periods. Dietary treatments were (1) an uncontaminated diet, (2) a contaminated diet (CD) mainly based on contaminated wheat with 1.6 mg/kg DON, (3) CD + 0.25% PB consisting of preservation components as major sources, antioxidants, microorganisms, and amino acids (AA), and (4) CD + 0.25% BEN. The AID and ATTD of dry matter, organic matter, crude protein, most minerals, and most AA were not affected by DON contamination. Dietary DON decreased the AID and ATTD of sodium (*p* < 0.05) but were restored by supplementing the PB product (*p* < 0.05). The AID of zinc was increased (*p* < 0.05) by dietary DON, but supplementing BEN decreased zinc digestibility (*p* < 0.05). The AID of Arg, Ile, Thr, and Asp was decreased (*p* < 0.05) by BEN addition. In conclusion, dietary DON affected the digestibility of some minerals but not AA in pigs. Supplemental BEN can negatively affect the nutrient digestibility of some minerals and AA in pigs. The addition of a PB product in pig diets can restore digestibility of sodium but not of other nutrients. Based on these observations, feed additives for alleviating DON effects on nutrient digestibility of pigs can be carefully selected by swine diet formulators.

## 1. Introduction

The occurrence of mycotoxins is one of the major concerns in the swine feed industry, and mycotoxins are generated by fungal activity as secondary metabolites [1,2]. These metabolites are commonly found in grains such as barley, corn, and wheat, which are major ingredients of swine diets, resulting in deleterious effects on pig growth. Deoxynivalenol (DON), generated by the *Fusarium* fungus, in pig diets typically induces vomiting and reduces feed intake in young pigs, which could cause subsequent growth retardation of pigs [3,4,5]. Additionally, dietary DON can affect the morphology of the intestinal mucosa [6], the immune system [7], and the health of pig organs [8].

There have been efforts to alleviate the adverse effects of dietary DON on the growth performance of pigs by supplementing feed additives to swine diets. Clay products are well known to adsorb mycotoxins in diets [8,9,10] and therefore are often used in swine diets. However, limited efficacy of clay products for adsorbing DON and compensating for the reduced growth performance of pigs has been reported in previous in vitro studies [9,10] and in vivo studies [11,12], respectively. Moreover, clay products potentially bind nutrients including minerals and amino acids (AA) in addition to mycotoxins because clays’ binding capacity is not specific [13]. While Li and Kim [14] and Van Le Thanh et al. [11] reported that a supplemental clay product did not affect phosphorus (P) and calcium (Ca) digestibility, Schell et al. [15] observed decreased absorption of P and Ca as well as other minerals including magnesium (Mg) and zinc (Zn) by supplemental clay product in pigs fed an aflatoxin-contaminated diet. Considering these inconsistent results among the literature, more information is needed on the influence of clays on mineral digestibility. Additionally, the influence of clays on AA digestibility is also unknown, in spite of the fact that clays potentially adsorb AA.

Similar to clays, blend products consist of preservatives, and various nutrients have shown to partially restore the growth retardation in pigs fed DON-contaminated diets in previous studies [11,16]. However, limited data for the effects of blend products containing preservatives and other nutrients on the digestibility of minerals and AA are available. To bridge these gaps, therefore, we aimed to determine the effects of supplementing a bentonite (BEN) product and a blend product containing preservatives and nutrients on the ileal digestibility of minerals and AA in pigs fed diets naturally contaminated with DON.

## 2. Materials and Methods

The experimental procedure was approved by the Institutional Animal Care and Use Committee at Konkuk University (Seoul, Republic of Korea, KU22221).

### 2.1. Animals, Diets, and Experimental Design

Twelve crossbred barrows (Landrace × Yorkshire) with a mean initial body weight (BW) of 69.4 kg (standard deviation = 3.5) surgically fitted with a T-cannula at the distal ileum based on the procedure described by Stein et al. [17] were individually housed in pens (2.0 × 2.0 m) equipped with a feeder and a nipple drinker. The animals were allotted to a triplicated 4 × 2 incomplete Latin square design with 4 dietary treatments and 2 periods to obtain 6 observations per treatment. A spreadsheet-based program was used to minimize potential carryover effects [18]. Uncontaminated wheat and DON-contaminated wheat were prepared (Table 1). The 4 dietary treatments were (1) an uncontaminated diet (UCD) mainly based on DON-uncontaminated wheat and soybean meal, (2) a contaminated diet (CD) mainly based on wheat contaminated with 1.64 mg/kg of DON and soybean meal, (3) CD + 0.25% of BEN, and (4) CD + 0.25% of a preservative blend (PB) consisting of preservation components, antioxidants, yeast components, microorganisms, and AA (Table 2 and Table 3). Each additive was supplemented to the CD at the expense of cornstarch. The experimental diets were formulated to meet or exceed the nutrient requirement estimates [19].

### 2.2. Feeding and Sample Collection

Based on the BW of each pig and the metabolizable energy concentration of the experimental diets, daily feed allowance for each pig was calculated at the beginning of each experimental period as 3 times the estimated energy requirement for maintenance (i.e., 197 kcal of metabolizable energy per kg BW^0.60^) [19]. The amount of feed allowance was divided into 2 equal meals and fed to pigs at 0800 and 1700 h. Water was freely available at all times.

After the 6-day adaptation period, fecal samples were collected for 24 h from 0900 on day 7. Ileal digesta samples were collected from 0900 to 1700 h on days 8 and 9. A plastic sample bag with wire was fixed to the T-cannula to collect the ileal digesta. The plastic sample bag was changed every 30 min or when the sample bag was filled with ileal digesta. The collected ileal digesta samples were immediately stored at −20 °C.

### 2.3. Chemical Analyses

The ileal digesta samples were freeze-dried and finely ground before analyses. Samples of ingredients, diets, ileal digesta, and feces were analyzed for dry matter (DM) based on the procedure suggested by Ahn et al. [20]. Crude protein (CP; method 990.03) and ash (method 942.05) were analyzed for ingredients, diets, ileal digesta, and feces according to the AOAC [21]. The ingredient, diet, and fecal samples were analyzed for gross energy (GE) using a bomb calorimeter (Parr 1261; Parr Instruments Co., Moline, IL, USA). The ingredients and the experimental diets were analyzed for ether extract (method 920.39), amylase-treated neutral detergent fiber (method 2002.04), acid detergent fiber (method 973.18), and starch (method 979.10). The experimental diets, ileal digesta, and feces were analyzed for Ca (method 978.02), P (method 964.06), and iron (Fe; method 937.03) according to the AOAC [21]. These samples were also analyzed for potassium (K), sodium (Na), magnesium (Mg), chlorine (Cl), and zinc (Zn) via inductively coupled plasma-optical emission spectrometry (method 985.01 A, B, and C) based on the AOAC [21]. The AA concentrations in the diets and ileal digesta were analyzed using the acid hydrolysis method (method 994.12), and sulfur-containing AA were analyzed by determining the Met sulfone and cysteic acid after cold performic acid oxidation overnight before hydrolysis (method 985.28), as suggested by the AOAC [21]. For analyzing chromium concentrations in the diet and ileal digesta, an ultraviolet-visible spectrophotometry (Optizen 2120UV, Mecasys Inc., Daejeon, Republic of Korea) was used. The concentrations of DON in the ingredient samples and experimental diets were determined using a high-performance liquid chromatography with an immunoaffinity chromatography column (ZORBAX Eclipse XDB-C18 column, 4.6 × 250 mm, 5 μM, Agilent, Santa Clara, CA, USA; [22]).

### 2.4. Calculations

The digestibility of nutrients was calculated based on the equation reported by Kong and Adeola [23]:

Digestibility, % = 100% − (Nutr_output_ ÷ Nutr_diet_) × (Cr_diet_ ÷ Cr_output_) × 100%

where Nutr_output_ is the concentration (%) of a nutrient in the ileal or fecal output, Nutr_diet_ is the concentration (%) of a nutrient in the experimental diet, Cr_diet_ is the Cr concentration (%) in the experimental diet, and Cr_output_ is the Cr concentration (%) in the ileal or fecal output.

### 2.5. Statistical Analyses

Experimental data were analyzed using the MIXED procedures of SAS. The model included the experimental diet as a fixed variable, whereas replication, animal within replication, and period within replication were random variables. Contrasts were conducted to test the DON effect (UCD vs. CD) and the feed additive effects (CD vs. BEN and CD vs. PB). Least squares means were calculated for each treatment and the means were separated using PDIFF option with Tukey’s adjustment. An individual pig was the experimental unit. The statistical significance and tendency were determined at *p* < 0.05 and 0.05 ≤ *p* < 0.10, respectively.

## 3. Results

The apparent ileal digestibility (AID) and apparent total tract digestibility (ATTD) of DM, organic matter (OM), and CP were not different in pigs fed the UCD and CD diets (Table 4). The AID and ATTD of DM, OM, and CP were not affected by supplemental BEN or the PB in the DON-contaminated diet.

The AID and ATTD of Na in the CD group were less (*p* < 0.05) than those in the UCD group (Table 5). However, the AID and ATTD of Na were increased (*p* < 0.05) by the supplemental PB in the DON-contaminated diet. The AID of Zn in the CD group was greater (*p* < 0.05) than that in the UCD group. However, the AID and ATTD of Zn were decreased (*p* < 0.05) by supplemental BEN in the DON-contaminated diet.

The AID of Arg, Ile, Thr, and Asp in pigs fed the BEN diet was less (*p* < 0.05) than that in pigs fed the CD (Table 6). In addition, the AID of His and Lys in pigs fed the BEN diet tended to be less (*p* < 0.10) than that in pigs fed the CD. The AID of AA except Met in pigs fed the PB diet did not differ from that in pigs fed the CD.

## 4. Discussion

Deoxynivalenol is one of most prevalent mycotoxins in swine diets and naturally occurs from *Fusarium* fungus [2]. Ingested DON by pigs causes inflammation and necrosis in the gastrointestinal tract of pigs, leading to a disturbance of gut function for nutrient absorption [6] and consequently, growth retardation of pigs [3,4,5]. Although a DON-contaminated diet can be supplemented with feed additives such as clay products and preservatives to alleviate negative influences of DON, the effects of supplementing these additives on ileal and total tract digestibility of minerals and ileal digestibility of AA are not well documented. Particularly, the potential adsorbing action of supplemental clays against minerals and AA is anticipated, but the regarding data for the negative effects on minerals and AA digestibility of pigs are scarce.

In the present study, the analyzed DON concentrations in diets containing DON-contaminated wheat were reasonably close to the expected values, except for the PB diet, which had a less analyzed DON concentration compared with the expected value. The low DON concentration in the PB diet is consistent with Van Le Thanh et al. [11], who reported a relatively low DON concentration in the diet. The low DON concentrations in DON-contaminated diets supplemented with the PB may be attributed to the influence of the preservative components in the PB that potentially inactivate DON [24,25]. These findings are supported by results from Dänicke et al. [26], who observed a dose-dependent reduction in DON concentration when supplementing sodium metabisulphite to the diets containing triticale kernels contaminated with 6.6 mg/kg of DON. The PB used in the present study also contained sodium metabisulphite.

The lack of difference in the apparent digestibility of DM and CP between the UCD and CD groups agrees with previous studies [24,27]. In contrast, Holanda et al. [28] reported decreases in the AID of DM, GE, and nitrogen in nursery pigs fed the DON-contaminated diet, but this may be due to the effects of other mycotoxins including fumonisin and zearalenone, as well as DON. Additionally, the response to dietary DON may vary depending on the growth stages of pigs. While dietary DON resulted in decreased DM and GE digestibility in nursery pigs [11,28], dietary DON did not affect DM or CP digestibility in growing pigs in the present work and other studies [24,27]. Older pigs may be less responsive to dietary DON compared with nursery pigs. The lack of effects of supplemental BEN or the PB on nutrient digestibility in the present work may be at least partially due to a lack of impaired digestibility of DM and CP by dietary DON.

The lack of the effects of dietary DON on the apparent digestibility of Ca and P in the present study is in agreement with Kwon et al. [12], who reported no effects of DON on Ca digestibility. In contrast, Bouchard et al. [24] reported an increase in AID of Ca and P in pigs fed a DON-contaminated barley diet (4.49 mg/kg of DON). In addition, Van Le Thanh et al. [11] also observed an increased retention-to-intake ratio of Ca and P by dietary DON at the level of 4.61 mg/kg in pigs. Dietary DON has been reported to reduce serum concentrations of Ca and P [29], likely leading to an increased absorption of Ca and P [30]. However, the reason for no response in Ca and P digestibility by dietary DON observed in the present work may be partially attributed to the relatively low dietary DON concentration compared with other studies.

The limited effect of supplementing BEN on the digestibility of Ca and P in pigs fed the DON-contaminated diet is consistent with previous studies [12,31,32], in which various clay minerals including BEN and maifanite were employed. These observations indicate that BEN may not largely adsorb Ca or P in the gastrointestinal tract of pigs. On the other hand, the observation that supplementing the PB did not affect the AID of Ca and P in the present work contrasts with Van Le Thanh et al. [11]. It is suggested that supplementation of the PB can alleviate the deterioration of health in pigs caused by the consumption of DON. Preservative components can inactivate DON in diets [24,25], and other components in the PB such as AA, vitamins, and microorganisms may improve pig health impaired by DON. However, the lack of effects of the supplemental PB on Ca and P digestibility remains unclear, although the relatively low DON concentration may partially explain the present results.

Very limited information is available for the effects of DON on mineral digestibility. The AID of K and Mg was not affected by dietary DON in the present study. Similarly, Zeebone et al. [33] fed a diet contaminated by fumonisin at 30 mg/kg to nursery pigs and found no changes in K and Mg digestibility due to fumonisin. In addition, aflatoxin B_1_ did not affect the absorption of Mg in pigs [15]. These results suggest that although mycotoxins in diets can negatively affect the growth performance of pigs, these toxins may not interrupt or stimulate the absorption of K or Mg. While the addition of BEN did not affect the apparent digestibility of Mg in the present study, Schell et al. [15] reported decreased Mg absorption by adding clays to diets regardless of DON contamination. The decrease in Mg absorption was also observed in sheep fed diets containing a clay product [34]. The reason for this discrepancy may include different sources of clay products, experimental conditions, and animals in the experiments.

For the effects of mycotoxins on Na absorption, inconsistent results have been reported in previous studies [13,31]. The increased Na absorption by consuming aflatoxin B_1_ in pigs was observed by Schell et al. [15], whereas no effect of dietary fumonisin on Na digestibility was observed by Zeebone et al. [33]. In the present work, the AID of Na was decreased by dietary DON. These observations suggest that the mechanism of the influence of mycotoxins on Na absorption in animal bodies is still unclear. However, different mycotoxins may have their own specific mechanism of interaction with Na. Additionally, as well as Na, the interaction of mycotoxins with the metabolism of K can also affect Na absorption in animal bodies because they serve as a part of the Na–K pump to balance anions intracellularly [19]. Clay products potentially degrade chlorinated compounds [35] and they might partially liberate Cl ions from the chlorinated compounds originating from diets and endogenous losses, increasing the absorption of Cl. Additionally, changes in Na metabolism and acid-base balance could affect the metabolism of Cl in pigs because Cl is associated with maintaining osmotic pressure in body fluids and acid-base balance with Na [19]. However, the reason for the lack of changes in the apparent digestibility of Cl due to BEN in the present study remains unclear. Further research is warranted to investigate the effects of dietary DON and supplementing mycotoxin-sequestering agents on macromineral digestibility.

The limited effects of dietary DON on the apparent digestibility of Fe of pigs observed in the present study agree with previous studies [15,36,37]. The increased digestibility of Zn by dietary DON observed in this study is in agreement with Yunus and Bohm [38], who reported that broilers fed diets contaminated with an increasing DON concentration (0, 1.7, and 12.2 mg/kg) for 5 weeks showed a linear increase in serum Zn concentration. A similar result was reported by Schell et al. [15], who fed an aflatoxin B_1_-contaminated diet to pigs and found an increase in Zn absorption. Exposure to mycotoxins has been suggested to alter the demands for Zn or the efficiency of Zn absorption in animal bodies [15,38]. As observed in the present work, the ability of clay products to decrease Zn digestibility or absorption is relatively clear. Schell et al. [15] reported that the Zn absorption was decreased by supplementing BEN in diets fed to pigs regardless of aflatoxin contamination. Chestnut et al. [34] also reported an approximately 50% decrease in Zn absorption due to supplementation of aluminosilicate in sheep. Because clay minerals have a large surface area and high structural charge, the divalent transition metals such as Zn can be adhered by clays [39], resulting in reduced absorption of Zn in animals.

Whereas no effect of dietary DON on the AID of all indispensable AA was observed in the present study, supplementing cultured DON to diets at the level of 10 mg/kg tended to decrease the digestibility of Lys, Thr, Trp, and Val compared with control diets fed to pigs [27]. These inconsistent results are likely due to the difference in dietary DON concentrations or the source of DON. High concentrations of dietary DON have the potential to destruct barriers of the intestinal epithelium, which has been confirmed by previous studies [40,41]. The decreased AID of some AA by supplementing BEN is likely because AA molecules can be adsorbed to the clay surface, which has been identified by previous studies [42,43]. It is likely that dietary AA, undigested AA, or both are adsorbed to clay products and excreted in the fecal form, leading to decreased digestibility. However, further research is warranted to identify the reasons for the effects of BEN on AA digestion and absorption in pigs.

## 5. Conclusions

The contaminated diets with 1.63 mg/kg deoxynivalenol fed to pigs did not affect ileal digestibility of dry matter, organic atter, and crude protein, which may be one of the potential reasons for the lack of improvement in the digestibility by clay or preservative blend products. Sodium digestibility was decreased by dietary deoxynivalenol but restored by the preservative blend product. Zinc digestibility was increased by dietary deoxynivalenol, but the addition of bentonite interrupted the digestion or absorption of zinc. Amino acid digestibility was not affected by dietary deoxynivalenol, but the digestibility of some amino acids was decreased by the supplemental bentonite product.

## Figures and Tables

**Table 1:** Analyzed chemical composition of uncontaminated wheat, naturally contaminated wheat with deoxynivalenol, and soybean meal, as-is basis.

Item, %	Uncontaminated Wheat	Contaminated Wheat	Soybean Meal
Dry matter	91.1	87.8	89.2
Gross energy, kcal/kg	3917	3813	4124
Crude protein	10.5	13.3	42.9
Ether extract	1.79	2.13	2.92
Neutral detergent fiber	13.8	12.3	9.51
Acid detergent fiber	3.20	3.25	4.83
Ash	1.62	1.73	6.51
Starch	69.4	67.4	- ^1^
Calcium	0.07	0.06	0.42
Phosphorus	0.25	0.34	0.65
Phytate-phosphorus	0.18	0.25	- ^1^
Deoxynivalenol, mg/kg	0.043	1.639	0.105

^1^ Not determined.

**Table 2:** Ingredient composition and chemical composition of experimental diets, as-is basis ^1^.

Items	UCD	CD	BEN	PB
Ingredient, %				
Uncontaminated wheat	86.1	-	-	-
Contaminated wheat ^2^	-	86.1	86.1	86.1
Soybean meal, 42.8% crude protein	9.5	9.5	9.5	9.5
Soybean oil	1.0	1.0	1.0	1.0
L-Lys·HCl, 78.8%	0.3	0.3	0.3	0.3
Ground limestone	1.7	1.7	1.7	1.7
Chromium oxide	0.5	0.5	0.5	0.5
Vitamin–mineral premix ^3^	0.3	0.3	0.3	0.3
Salt	0.3	0.3	0.3	0.3
Corn starch	0.25	0.25	-	-
Bentonite	-	-	0.25	-
Preservative blend	-	-	-	0.25
Analyzed chemical composition				
Dry matter, %	91.2	88.2	88.2	88.3
Gross energy, kcal/kg	3893	3799	3802	3796
Crude protein, %	13.3	15.6	15.5	15.5
Ether extract, %	3.2	3.5	2.9	3.2
Amylase-treated neutral detergent fiber, %	13.7	11.9	11.2	11.5
Acid detergent fiber, %	3.30	3.41	3.18	3.36
Ash, %	4.62	4.73	4.87	4.94
Calcium, %	0.74	0.71	0.67	0.77
Phosphorus, %	0.25	0.33	0.33	0.35
Potassium, %	0.41	0.42	0.41	0.47
Sodium, %	0.13	0.10	0.11	0.17
Magnesium, %	0.11	0.13	0.13	0.14
Chloride, %	0.29	0.32	0.28	0.35
Iron, mg/kg	213	217	273	252
Zinc, mg/kg	64.5	87.5	66.0	97.5
Deoxynivalenol, mg/kg	0.036	1.628	1.564	0.636

^1^ UCD = uncontaminated diet; CD = contaminated diet with 1.63 mg/kg deoxynivalenol; BEN = CD + 0.25% of bentonite; PB = CD + 0.25% of preservative blend. ^2^ The contaminated wheat contained 1.64 mg/kg of deoxynivalenol (Table 1). ^3^ Provided the following quantities per kilogram of complete diet: vitamin A, 20,000 IU; vitamin D_3_, 3000 IU; vitamin E, 100 mg; vitamin K, 2.5 mg; thiamin, 5 mg; riboflavin, 7.5 mg; pyridoxine, 3.5 mg; vitamin B_12_, 0.03 mg; pantothenic acid, 22.5 mg; folic acid, 0.5 mg; niacin, 27.5 mg; biotin, 0.5 mg; Cu, 11 mg as copper sulfate; Fe, 62.8 mg as iron sulfate; I, 0.97 mg as calcium iodate; Mn, 10.7 mg as manganese sulfate; Zn, 29.8 mg as zinc sulfate; Co, 0.01 mg as cobaltous carbonate.

**Table 3:** Amino acid concentrations of experimental diets, as-is basis ^1^.

Items, %	UCD	CD	BEN	PB
Indispensable amino acid				
Arg	0.71	0.70	0.59	0.70
His	0.32	0.33	0.30	0.35
Ile	0.43	0.46	0.40	0.47
Leu	0.87	0.92	0.81	0.93
Lys	0.92	0.81	0.76	0.73
Met	0.19	0.22	0.24	0.26
Phe	0.67	0.70	0.62	0.71
Thr	0.55	0.56	0.48	0.53
Val	0.62	0.65	0.59	0.65
Dispensable amino acid				
Ala	0.48	0.48	0.43	0.48
Asp	1.01	0.98	0.80	0.96
Cys	0.26	0.29	0.31	0.30
Glu	3.00	3.45	3.21	3.61
Gly	0.54	0.55	0.51	0.55
Pro	1.10	1.27	1.25	1.40
Ser	0.66	0.70	0.68	0.72
Tyr	0.47	0.47	0.43	0.52

^1^ UCD = uncontaminated diet; CD = contaminated diet with 1.63 mg/kg deoxynivalenol; BEN = CD + 0.25% of bentonite; PB = CD + 0.25% of preservative blend.

**Table 4:** Effects of feed additives in deoxynivalenol-contaminated diets on apparent ileal and total tract digestibility of nutrients ^1,2^.

Item, %	UCD	CD	BEN	PB	SEM	*p*-Value for Contrast		
						UCD vs. CD	CD vs. BEN	CD vs. PB
Apparent ileal digestibility								
Dry matter	69.0	72.3	72.3	73.4	2.1	0.277	0.993	0.702
Organic matter	71.0	74.7	74.9	75.6	2.0	0.204	0.953	0.742
Crude protein	72.9	76.7	75.3	77.6	2.0	0.183	0.609	0.736
Apparent total tract digestibility								
Dry matter	90.0	89.4	89.2	88.6	0.7	0.516	0.807	0.414
Organic matter	91.4	91.0	90.9	90.2	0.7	0.586	0.963	0.354
Crude protein	86.9	88.0	87.8	87.6	1.4	0.533	0.892	0.791

^1^ Data are least squares means of 6 observations. ^2^ UCD = uncontaminated diet; CD = contaminated diet with 1.63 mg/kg deoxynivalenol; BEN = CD + 0.25% of bentonite; PB = CD + 0.25% of preservative blend.

**Table 5:** Effects of feed additives in deoxynivalenol-contaminated diets on apparent ileal and total tract digestibility of minerals ^1,2^.

Item, %	UCD	CD	BEN	PB	SEM	*p*-Values for Contrast		
						UCD vs. CD	CD vs. BEN	CD vs. PB
Apparent ileal digestibility								
Calcium	51.5	49.4	47.5	55.3	5.1	0.749	0.770	0.380
Phosphorus	35.5	37.7	39.6	45.2	4.1	0.639	0.694	0.129
Potassium	72.1	75.9	71.2	78.6	3.7	0.458	0.342	0.583
Sodium	−230.1	−352.4	−344.5	−130.0	32.9	0.008	0.846	<0.001
Magnesium	11.3	12.3	11.8	21.4	4.6	0.875	0.935	0.150
Chloride	72.0	64.7	62.2	64.2	7.8	0.432	0.798	0.965
Iron	5.0	6.0	14.4	14.4	4.7	0.852	0.127	0.127
Zinc	−1.1	21.1	1.7	26.2	3.3	<0.001	0.001	0.290
Apparent total tract digestibility								
Calcium	70.9	68.2	63.9	69.3	3.2	0.483	0.228	0.770
Phosphorus	54.8	58.3	56.1	57.1	2.5	0.293	0.471	0.697
Potassium	74.6	74.1	77.6	76.9	3.1	0.908	0.341	0.458
Sodium	83.2	74.5	67.5	83.6	3.7	0.048	0.106	0.039
Magnesium	47.0	48.0	43.7	47.8	2.7	0.755	0.151	0.944
Chloride	95.2	94.3	94.6	95.0	0.9	0.366	0.788	0.475
Iron	24.1	20.6	28.8	23.7	5.6	0.594	0.203	0.630
Zinc	7.6	11.2	−11.2	8.3	5.5	0.623	0.010	0.681

^1^ Data are least squares means of 6 observations. ^2^ UCD = uncontaminated diet; CD = contaminated diet with 1.63 mg/kg deoxynivalenol; BEN = CD + 0.25% of bentonite; PB = CD + 0.25% of preservative blend.

**Table 6:** Effects of feed additives in deoxynivalenol-contaminated diets on apparent ileal digestibility of amino acids ^1,2^.

Item, %	UCD	CD	BEN	PB	SEM	*p*-Value for Contrast		
						UCD vs. CD	CD vs. BEN	CD vs. PB
Indispensable amino acid								
Arg	84.2	84.0	79.8	85.1	1.5	0.886	0.043	0.557
His	81.6	82.3	78.5	84.1	1.6	0.752	0.088	0.415
Ile	79.3	80.0	75.4	81.9	1.5	0.709	0.040	0.369
Leu	81.2	81.9	78.1	83.4	1.7	0.779	0.135	0.515
Lys	84.4	82.6	79.0	81.8	1.5	0.358	0.087	0.720
Met	77.8	80.5	82.3	84.9	1.7	0.276	0.463	0.086
Phe	80.8	81.6	78.3	83.2	1.7	0.734	0.192	0.514
Thr	74.5	74.5	67.9	74.4	2.0	0.994	0.027	0.963
Val	76.3	77.6	73.3	79.3	2.0	0.661	0.146	0.538
Dispensable amino acid								
Ala	70.3	71.2	64.1	72.1	2.7	0.815	0.074	0.802
Asp	76.0	74.1	64.6	75.1	2.1	0.503	0.004	0.740
Cys	72.8	74.4	76.0	75.9	2.2	0.617	0.615	0.639
Glu	83.7	86.7	86.0	87.8	1.6	0.196	0.737	0.622
Gly	61.5	65.0	65.0	64.0	4.9	0.609	0.994	0.879
Pro	79.4	83.7	83.1	85.6	1.6	0.054	0.761	0.336
Ser	77.3	78.2	76.0	79.6	1.6	0.666	0.325	0.557
Tyr	75.7	77.2	73.4	79.7	1.7	0.539	0.134	0.296

^1^ Data are least squares means of 6 observations. ^2^ UCD = uncontaminated diet; CD = contaminated diet with 1.63 mg/kg deoxynivalenol; BEN = CD + 0.25% of bentonite; PB = CD + 0.25% of preservative blend.

## Data Availability

The data presented in the current work are available.
